# Lower Ratio of Liver Volume and Body Weight Is a Negative Predictor of Survival after Transjugular Intrahepatic Portosystemic Shunt

**DOI:** 10.3390/jpm11090903

**Published:** 2021-09-09

**Authors:** Philipp Schindler, Arne Riegel, Dennis Görlich, Jeanette Köppe, Leon Louis Seifert, Max Masthoff, Miriam Maschmeier, Christian Wilms, Max Seidensticker, Michael Köhler, Jonel Trebicka, Hauke Heinzow, Moritz Wildgruber

**Affiliations:** 1 Clinic for Radiology, University Hospital Muenster, 48149 Muenster, Germany; arne.riegel@ukmuenster.de (A.R.); Max.Masthoff@ukmuenster.de (M.M.); michael.koehler@ukmuenster.de (M.K.); Moritz.Wildgruber@med.uni-muenchen.de (M.W.); 2 Institute of Biostatistics and Clinical Research, University Hospital Muenster, 48149 Muenster, Germany; dennis.goerlich@ukmuenster.de (D.G.); Jeanette.Koeppe@ukmuenster.de (J.K.); 3 Department of Gastroenterology and Hepatology, University Hospital Muenster, 48149 Muenster, Germany; leonlouis.seifert@ukmuenster.de (L.L.S.); Miriam.Maschmeier@ukmuenster.de (M.M.); christian.wilms@ukmuenster.de (C.W.); h.heinzow@bk-trier.de (H.H.); 4 Department of Medicine I, Bruederkrankenhaus Trier, 54292 Trier, Germany; 5 Department of Radiology, University Hospital, LMU Munich, 81377 Munich, Germany; max.seidensticker@med.uni-muenchen.de; 6 Department of Internal Medicine I, University Hospital Frankfurt, 60590 Frankfurt, Germany; jonel.trebicka@kgu.de

**Keywords:** transjugular intrahepatic portosystemic shunt, transplant-free survival, liver cirrhosis, liver volume

## Abstract

Transjugular intrahepatic portosystemic shunt (TIPS) is the most effective measure to treat complications of portal hypertension. However, liver function may deteriorate after TIPS. Predictors of liver function and outcome after TIPS are therefore important for management of TIPS patients. The study aimed to evaluate the impact of liver volume on transplant-free survival (TFS) after TIPS, as well as the evolution of liver volume and its relationship with liver function after TIPS. A retrospective analysis of all consecutive patients who underwent TIPS in a tertiary care university liver center between 2012 and 2017 (*n* = 216) was performed; *n* = 72 patients with complete prior and follow-up (FU) computed tomography (CT) imaging studies were included in the study. Volumetry of the liver was performed by a semi-automatic 9-lobe image segmentation algorithm at baseline and FU (FU 1: 90–180 d; FU 2: 180–365 d; FU 3: 365–545 d; FU 4: 545–730 d; FU 5: >730 d). Output variables were total liver volume (TLV, cm^3^), left liver volume (LLV, cm^3^), right liver volume (RLV, cm^3^) and TLV/body weight ratio. CT derived liver volumes were correlated with liver function tests, portosystemic pressure gradient (PPG) measurements and survival. To assess predictors of liver volume change over time we fitted linear mixed models. Kaplan–Meier analysis was performed and validated by matched pair analysis followed by Cox regression to determine independent prognostic factors for survival. The median TLV at baseline was 1507.5 cm^3^ (773.7–3686.0 cm^3^). Livers with higher baseline liver volumes and larger TLV/weight ratios retained their volume after an initial loss while smaller livers continuously lost volume after TIPS. At the first follow-up period (90–180 d post-TIPS) lower liver volumes and TLV/weight ratios were associated with higher bilirubin levels. Within the final multivariable model containing time (days since TIPS), baseline INR and baseline TLV, the average loss of liver volume was 0.74 mL per day after TIPS. Twelve-month overall transplant-free survival was 89% and median overall TFS was 33 months. The median TFS for a baseline TLV/body weight ratio > 20 was significantly higher compared with ≤20 (40.0 vs. 27.0 months, *p* = 0.010) while there were no differences regarding the indication for TIPS or etiology of liver disease in the matched pair analysis. Lower TLV/weight ratios before TIPS were associated with shorter TFS and should therefore be critically considered when selecting patients for TIPS. In addition, this study provides first evidence of an effect of TIPS on subsequent liver volume change and associated liver function.

## 1. Introduction

Transjugular intrahepatic portosystemic shunt (TIPS) is an established treatment in the management of decompensated liver cirrhosis [1,2]. Technical progress has resulted in improved patency rates and reduced TIPS-related complications [1,3,4,5]. In terms of clinical outcome, TIPS significantly prolongs transplant-free survival in advanced cirrhosis [4]. In contrast, creating a portosystemic shunt alters the intrahepatic hemodynamics and results in decreased liver perfusion with possible impairment of liver function and progression to liver failure [5]. In cirrhotic patients, loss of liver volume leads to deterioration of liver function and is a poor prognostic factor in cirrhosis [6,7]. However, liver volume has not yet been extensively studied as a potential variable being suitable for patient selection for TIPS [6]. Despite a thorough patient evaluation proceeding for TIPS, including assessment of the Model of End-Stage Liver Disease (MELD), Freiburg index of post-TIPS survival (FIPS), Child–Pugh score and hepatic encephalopathy, there are still challenges regarding the appropriate workup for TIPS patients [8]. The assessment of the liver volume using pre-procedural imaging may have a predictive value for post-TIPS liver function and outcome and hence might be a simple tool in patient selection to optimize the balance of detriments and benefits achieved by TIPS [6,9,10,11].

CT volumetry is increasingly established in current clinical practice to accurately determine the liver volume [12]. It is useful in the pre-operative planning of major resection or living donor liver transplantation where the technical and clinical success depend on the future remnant liver volume or liver graft size in transplantation [12,13,14]. However, there are only limited data regarding the imaging-based assessment of liver volume in the patient selection for TIPS and follow-up of TIPS patients [6,15]. 

The aim of our study was to evaluate the impact of liver volume on transplant-free survival (TFS) after TIPS. Additionally, we aimed to analyze the evolution of liver volume and its relationship with liver function after TIPS.

## 2. Results

### 2.1. Patient Characteristics

Seventy-two patients with complete baseline and follow-up data were included in the study (median age, y: 60 (21–81); *n* = 40 male, *n* = 32 female; Table 1). The most frequent etiology of liver disease was alcohol abuse. Liver disease was mostly frequently graded as Child–Pugh B. Mean MELD-score pre-TIPS was 11.7 (SD: 6.0). Refractory ascites was the most frequent indication of TIPS insertion.

### 2.2. Development of Liver Function and Liver Volume following TIPS Insertion

Liver function parameters and portal pressure gradient (PPG) were recorded at baseline before TIPS and modelled as repeated measurements after TIPS procedure at five follow-up (FU) periods. As shown in Table 2, there were significant alterations over time: MELD-score (*p* = 0.0224), bilirubin (*p* = 0.0008), International Normalized Ratio (INR, *p* = 0.0125), lactate dehydrogenase (LDH, *p* = 0.0004), quick (*p* = 0.0057) and sodium (*p* = 0.0003). 

**Figure 1:**
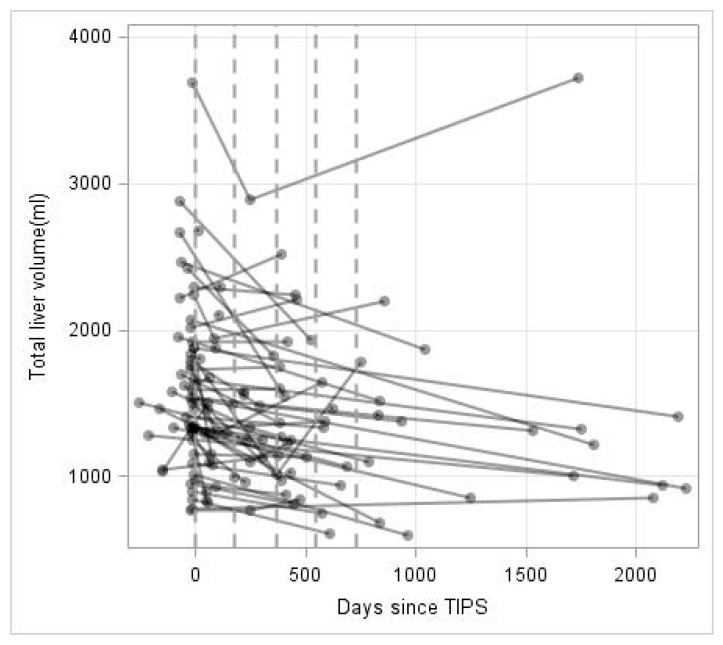
Total liver volume (mL) over time (in days). Repeated measurements are connected by lines for each patient. Discretized assessment periods are marked by dashed vertical lines.

The median pre-procedural TLV was 1507.5 cm^3^ (773.7–3686.0 cm^3^). Figure 1 shows the liver volume data over time of all patients included. The dashed lines indicate the follow-up periods used for the descriptive analysis of the data indicating an overall TLV loss over time. Repeated volume measurements were analyzed for TLV, LLV, RLV and TLV/weight ratio. As shown in Table 3, TLV (*p* < 0.0001), LLV (*p* = 0.0102), RLV (*p* < 0.0001) and TLV/weight ratio (*p* < 0.0001) revealed significant decreases over time. The TLV reduced on average by 10.7% within the first 365 days. LLV and RLV reduced by 14.1% and 9.5%, respectively. The TLV/weight ratio decrease by 15.8% within the first 365 days after TIPS.

Next, we aimed to analyze liver function after TIPS in relation to liver volume. As shown in Table 4, a correlation analysis of total liver volume at the first follow-up period (FU1: 90–180 d) showed a negative correlation between TLV and bilirubin (Spearman correlation: −0.44, *p* = 0.03839), indicating that lower liver volume is associated with higher bilirubin levels. All other parameters could not be proved to be correlated with TLV. Similarly, TLV/weight ratio and bilirubin were correlated (Spearman correlation: −0.46, *p* = 0.04249), while TLV/weight ratio and the MELD-score showed a correlation descriptively (Spearman correlation: −0.42, *p* = 0.0649). None of the remaining parameters showed a significant correlation with TLV/weight ratio.

### 2.3. Analysis of Predictors for the Development of Liver Volume after TIPS

**Figure 2:**
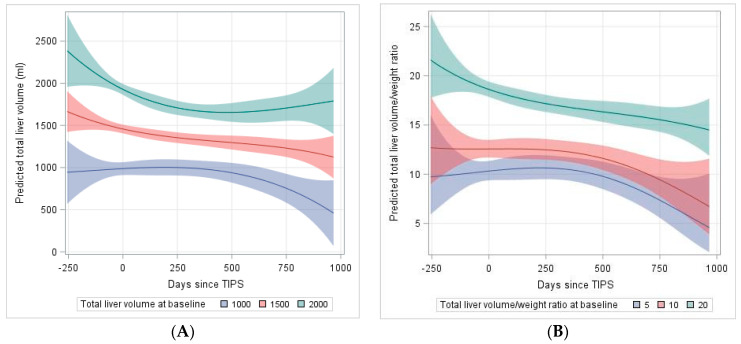
Explorative model of (**A**) total liver volume pre-TIPS to 1000 days after TIPS dependent on baseline liver volume (**B**) total liver volume (TLV)/weight ratio pre-TIPS to 1000 days after TIPS dependent on baseline TLV/weight ratio. TIPS was applied at day 0. The underlying models are polynomial models of 3rd degree including interactions with baseline volume (volume/weight ratio).

Based on follow-up liver volume data indicating an overall TLV loss over time, we fitted linear mixed models which predict the development of TLV following TIPS. As shown in Table 5, this model reveals a loss in TLV over time depending on the preexisting volume before TIPS (coefficient = −0.00143, interaction *p* = 0.0251). The higher order (Time^3^ × volume) term also shows a minor but significant contribution to the volume over time (coefficient = −14 × 10^−11^, interaction *p* ≤ 0.0001). Based on this model, Figure 2A presents the predicted means for the TLV over time and its course for three selected baseline volumes (1000 mL, 1500 mL, 2000 mL) over the first 1000 days after TIPS. While livers with higher baseline volumes retain their volume after initial loss, smaller livers (below 1500 mL) continue to lose volume over time. Similarly, we fitted a polynomial model for the TLV/weight ratio. Here, the time × TLV/weight ratio (before TIPS) does not show a significant decline over time, while the higher order terms indicate a decrease of TLV/weight ratio over time (Table 6) as shown in Figure 2B for three selected baseline ratios (5, 10, 20) over the first 1000 days after TIPS.

Finally, we aimed to develop a multivariable linear mixed model to stratify TIPS patients based on their baseline TLV and the expectable development of TLV following TIPS using routinely available clinical variables. From all available baseline variables, candidates were selected from the univariate analysis (*p* < 0.2). Here, age (interaction with time *p* = 0.0095), Alanine Aminotransferase (ALT, interaction *p* = 0.0479), INR (interaction *p* = 0.068), and baseline TLV (*p* < 0.0001) emerged as prominent covariates. A multivariable model was learned by backward selection by Schwarz Bayesian Criterion (SBC). The final multivariable model contains time (days since TIPS placement), INR before TIPS, the interaction between INR before TIPS and time, and finally baseline TLV. All factors were significantly associated with TLV after TIPS (all *p* < 0.02, Table 7). Within this multivariable model the average loss of liver volume is −0.74 mL/day (95%CI: −1.09 mL/day to −0.40 mL/day).

### 2.4. Survival Analysis Regarding Baseline Liver Volumes

**Figure 3:**
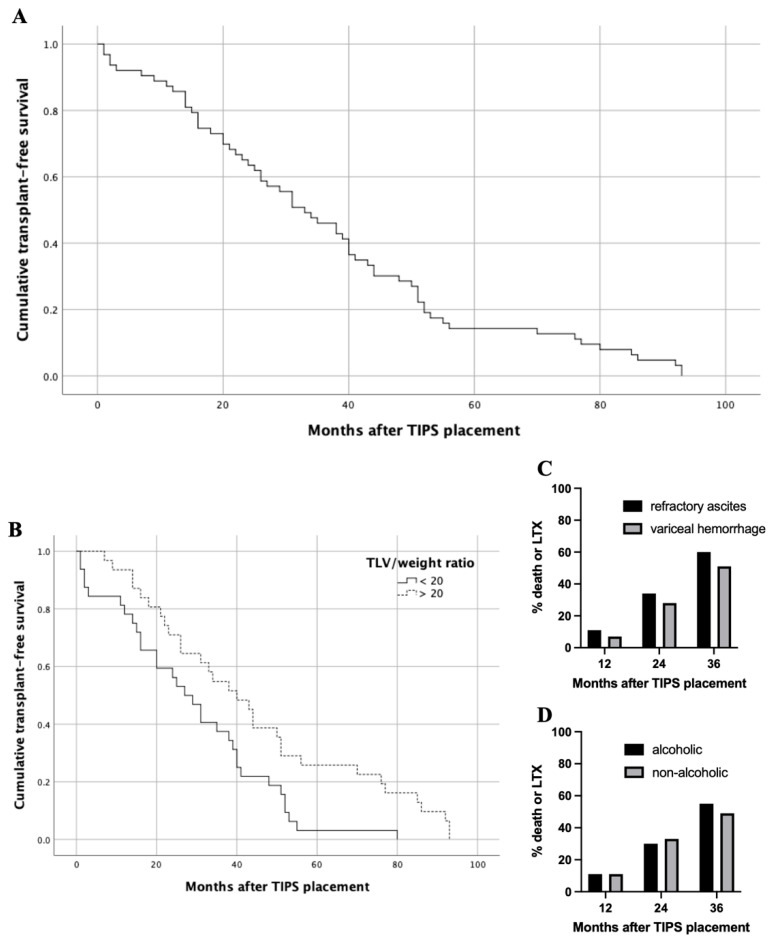
Transplant-free survival (TFS) of entire patient cohort and subgroup analysis. (**A**) Twelve-month overall TFS was 89% and median overall TFS was 33 months. (**B**) Median TFS for a baseline TLV/body weight ratio > 20 was significantly higher compared with <20 (40.0 vs. 27.0 months, *p* = 0.010). (**C**) In matched pair analysis regarding indication for TIPS; death or LTX occurred in 11%, 34% and 60% of patients with refractory ascites 12, 24 and 36 months after TIPS, respectively. Death or LTX occurred in 7%, 28% and 51% of patients with variceal hemorrhage 12, 24 and 36 months after TIPS, respectively; (each *p*-value at 12, 24 and 36 months > 0.05). (**D**) In matched pair analysis regarding etiology of liver cirrhosis; death or LTX occurred in 11%, 30% and 55% of patients with alcoholic history 12, 24 and 36 months after TIPS, respectively. Death or LTX occurred in 11%, 33% and 49% of patients with non-alcoholic history 12, 24 and 36 months after TIPS, respectively; (each *p*-value at 12, 24 and 36 months > 0.05). Abbreviations: TIPS, transjugular intrahepatic portosystemic shunt; LTX, liver transplantation.

Twelve-month TFS was 89% and median TFS was 33 months (Figure 3A). The median TFS for a baseline TLV/weight ratio > 20 was significantly higher compared with ≤ 20 (40.0 vs. 27.0 months, *p* = 0.010; Figure 3B). There were no significant differences in the median survival regarding baseline TLV, LLV and RLV. Due to significant differences in the TFS regarding the TLV/weight ratio, we performed a matched pair analysis to validate the predictive value. Matched patients were sub-divided based on indication for TIPS (refractory ascites vs. variceal hemorrhage) or etiology of liver disease (alcoholic vs. non-alcoholic). Here, subgroup survival analysis revealed no significant differences concerning indication for TIPS or the etiology of liver cirrhosis in the matched pair cohort (Figure 3C,D).

Model selection for multivariate analysis of baseline liver volume parameters on survival was done by forward and backward elimination with a threshold of *p* < 0.15 for inclusion in the model. A baseline TLV/weight ratio > 20 was found to be an independent parameter with a significant influence on survival (*p* = 0.013).

## 3. Discussion

Various models have been developed to predict survival after TIPS, while the MELD-score is one of the most commonly used in clinical routine [5,6,16,17,18,19]. Careful patient selection proceeding for TIPS continues to be a clinical challenge as some patients may develop post-TIPS liver failure despite initially preserved liver function as determined by laboratory data [6]. TIPS insertion alters the intrahepatic hemodynamics while the reduced portal blood supply may affect the reserve volume and function of the liver [20]. In cirrhotic patients, the decreasing liver volume results in an impairment of liver function while a smaller liver is a poor prognostic factor in cirrhosis [5,8]. Although imaging-based assessment of the liver volume is increasingly established in the workup before major liver resection or living donor liver transplantation, there is only limited data about a possible predictive value of the liver volume on post-TIPS morbidity and mortality [6,7,12,13,15]. Further on, liver volume can thereby be determined non-invasively in a stable and reproducible manner by cross sectional imaging with an accuracy of up to 5%, whereas the parameters for calculating the established Child- or MELD-scores tend to vary to a higher degree [6,21].

Within this study, we included *n* = 72 patients who underwent TIPS with a median pre-procedural TLV of 1507.5 cm^3^. In a recent study, however with older data, Lopera et al. performed a retrospective review of 80 patients, analyzing the effect of liver volume on morbidity and mortality after TIPS [6]. In a comparable manner, Lopera et al. performed a software-based semi-automatic CT- or MRI-based assessment of TLV before TIPS with comparable baseline data (median TLV: 1420 cm^3^), while they did not provide an analysis of liver volume subgroups and liver volume development [6]. Within their cohort, Lopera et al. were not able to identify a possible impact of pre-TIPS TLV on self-defined major adverse events (hepatic encephalopathy requiring hospital admission, increase in >2 points in MELD-score > 18 points, need for emergent liver transplantation and/or death) [6]. Within the patient cohort of Lopera et al., *n* = 15 (19%) deaths occurred within 6 months after TIPS, whereas no significant associations were found when modelling TLV in terms of death [6]. In contrast, in our study only *n* = 5 (7%) deaths occurred within the 6-month follow-up. We were able to show that median TFS for a baseline TLV/weight ratio > 20 was significantly higher compared with ≤20 (40.0 vs. 27.0 months). These results were validated in a matched pair analysis without differences regarding the indication for TIPS and etiology of liver disease. Beyond that baseline TLV/weight ratio > 20 was found to be an independent parameter with a significant influence on survival.

In another single-center study, Cohen et al. described *n* = 68 patients with cross sectional imaging (CT/MRI) liver volumetry prior to TIPS placement [15]. In contrast to our study, liver volume assessment was based on linear hepatic measurements, which is not as accurate as the segmentation method used in our study. The overall 30-day mortality of 13.2% was notably raised compared to our cohort (30-day mortality: 3%) [15]. Cohen et al. were able to delineate a smaller baseline liver volume as a predictive factor for increased 30-day mortality after TIPS creation [15]. 

To the best of our knowledge there are no comparable studies analyzing the development of liver volume after TIPS. Based on follow-up liver volume data after TIPS, we fitted a model which predicts the development of the TLV over time. Livers with higher baseline volumes or larger TLV/weight ratios retain their volume after initial loss, suggesting that in these cases the liver still seems to have some compensatory capacity after initial volume loss. In contrast, smaller livers (below 1500 mL) or lower TLV/weight ratios (below 20) continue to lose volume over time and revealed higher bilirubin levels in the follow-up, indicating that the small liver is more likely to have their reserve volume and thus their synthesis function compromised. Thus, baseline liver volume and TLV/weight ratio should be considered as important parameters for patient selection in TIPS. In particular, patients with a low liver volume (<1500 mL) should be evaluated particularly carefully, as low liver volume is potentially associated with poorer outcome as shown here for the baseline TLV/weight ratio < 20. 

Moreover, we were able to develop a model to stratify TIPS patients based on their baseline TLV and the expectable development of TLV following TIPS using routinely available clinical variables. Before integration of this mathematical model prediction into routine workflows, further analyses are needed to establish a more standardized approach in the future. Nevertheless, combining a CT liver volume score as a simple, non-invasive tool with the assessment of the MELD, FIPS and Child–Pugh score and/or hepatic encephalopathy might optimize patient selection for TIPS.

This study is limited by its retrospective study design, including a potential selection bias. Future studies should include our suggested CT volumetry protocol to validate the prospective value of TLV in patients undergoing TIPS.

In conclusion, this study provides first evidence of an impact of TIPS placement on subsequent liver volume development and accompanied liver function. Baseline TLV/weight ratio before TIPS has a substantial effect on the transplant-free survival which has to be considered when selecting patients for shunt procedures.

## 4. Materials and Methods

### 4.1. Study Design and Patient Selection

The study was performed as a retrospective single-center observational trial in a tertiary care university liver center. The study was conducted in accordance with the Declaration of Helsinki (as revised in 2013) and the study protocol was approved by the local ethics committee (No.: 2016-046-f-S). Due to the retrospective character of this trial, informed consent was waived. The clinical data of all patients who underwent TIPS between 2012 and 2017 (*n* = 216) were collected; *n* = 144 patients had to be excluded due to missing baseline or follow-up CT imaging (*n* = 134) or liver transplantation (*n* = 10) before first routine follow-up following TIPS. Finally, *n* = 72 patients were included in the study. 

### 4.2. TIPS Procedure

TIPS creation was performed according to standard operating procedures under general anesthesia. Single-shot antibiosis was performed by administration of 2 g ceftriaxone intravenously. All TIPS procedures were performed using PTFE-covered Stents (Viatorr, Gore Inc., Flagstaff, AZ, USA). Initially, shunts were sized to 8 mm diameter; in cases of insufficient reduction of the portosystemic gradient (>12 mmHg) the shunt lumen was further increased to 9 and 10 mm. Afterward, TIPS patients routinely were put on 100 mg acetylsalicylate acid daily for life.

### 4.3. Data Collection and Follow-Up

All patient and procedural data including laboratory parameters, invasive portosystemic pressure gradient measurements and follow-up/survival data were retrospectively acquired from the electronic patient records as well as from the Picture Archiving and Communications System (PACS). The electronic patient records were reviewed for baseline patient characteristics including age, sex, etiology of liver disease, Child’s grade and indication for TIPS insertion. Laboratory data and invasive portosystemic pressure gradient measurements were obtained, and MELD-score was calculated at baseline (*n* = 72) and 5 follow-up (FU) periods after TIPS procedure (FU 1: 90–180 d, *n* = 32; FU 2: 180–365 d, *n* = 36; FU 3: 365–545 d, *n* = 26; FU 4: 545–730 d, *n* = 10; FU 5: >730 d, *n* = 19). Survival data was collected until death, until liver transplantation or until end of follow-up. Transplant-free survival (TFS) was defined as survival free of death of any cause or until liver transplantation.

### 4.4. Imaging-Based Volumetry of the Liver

Volumetry of the liver was performed on contrast enhanced CT using commercial software (IntelliSpace Portal, Philips, Best, The Netherlands). This algorithm calculates the liver volume based on semi-automatic 9-lobe image segmentation [22]. Output variables were total liver volume (TLV, cm^3^), left liver volume (LLV, cm^3^) including segments I-III, right liver volume (RLV, cm^3^) including segments IV-VIII, and TLV/weight ratio calculated as ($\frac{TLV,\;\;\;cm^{3}}{body\;weight,\;\;\;kg}$).

### 4.5. Statistics

Demographic parameters and liver volumes at baseline and during follow-up were reported as total number and percentage, mean and standard deviation or median and range or 95% confidence interval (CI), as appropriate. Analysis of variance was used to analyze the statistical significance for two paired/unpaired groups or multiple groups. Spearman’s correlation coefficient was used to assess correlation between liver volume and liver function. To assess predictors of liver volume over time we fitted linear mixed models. Each model contained the potential predictor variables as main effect and the interaction between predictor and assessment time. Repeated measurements were modelled in the random effect by using a power covariance structure. At first, univariate interaction models were fitted for each predictor variable. In a second step a multivariable model was fitted, using the predictor variables with a Wald-test *p*-value < 0.2 within a backward selection procedure. Kaplan–Meier analysis and log-rank test were performed to analyze survival. For validation analysis we performed a matched pair analysis. Propensity score matching was performed via logistic regression to create a propensity score for each patient, entering the following variables: age, sex, MELD-score and TLV/weight ratio. Matched patients were sub-divided based on indication for TIPS (refractory ascites vs. variceal hemorrhage) or etiology of liver disease (alcoholic vs. non-alcoholic) with a ratio of 2:1 (*n* = 28:*n* = 14) or 1:1 (*n* = 36:*n* = 36), respectively. A matched pair between the subgroups was obtained by use of nearest-neighborhood-matching using a caliper width of 0.2 without replacement as described elsewhere [23]. Model selection for multivariate analysis of baseline liver volumes on survival was done by using Cox regression with forward and backward elimination with a threshold of *p* < 0.15 for inclusion in the model; *p*-values < 0.05 were considered to be statistically significant. Statistical analysis was performed using the SPSS Statistics version 26 (SPSS Inc., Chicago, IL, USA) and SAS/STAT software version 9.4 (SAS Institute Inc., Cary, NC, USA). 

## Figures and Tables

**Table 1:** Patient characteristics.

	No. (%), Median (Range) or Mean (SD)
Total	72 (100)
Sex	
male	40 (55.6)
female	32 (44.4)
median age (range), y	60 (21–81)
etiology of liver disease	
alcoholic	36 (50.0)
viral	9 (12.5)
NAFLD	8 (11.1)
other	19 (26.4)
Child’s grade	
A	14 (19.4)
B	47 (65.3)
C	11 (15.3)
Mean MELD-score pre-TIPS (SD)	11.7 (6.0)
indication for TIPS	
refractory ascites	54 (75.0)
variceal hemorrhage	14 (19.4)
other	4 (5.6)

Abbreviations: MELD, Model of End-Stage Liver Disease; NAFLD, non-alcoholic fatty liver disease; SD, standard deviation; TIPS, transjugular intrahepatic portosystemic shunt.

**Table 2:** Baseline and follow-up laboratory data and pressure measurements regarding TIPS insertion (mean (SD)).

	Baseline *n* = 72	FU 1	FU 2	FU 3	FU 4	FU 5	*p*-Value *
		90–180 d *n* = 32	180–365 d *n* = 36	365–545 d *n* = 26	545–730 d *n* = 10	>730 d *n* = 19	
MELD-score	12.08 (6.01)	12.44 (7.79)	12.31 (3.89)	13.89 (6.33)	15.22 (6.16)	9.52 (3.28)	0.0224
Albumin, g/dL	3.55 (3.99)	2.81 (0.68)	3.2 (0.39)	3.13 (0.72)	3.39 (0.71)	3.53 (0.61)	0.8792
Bilirubin, mg/dL	1.72 (1.01)	2.63 (2.91)	2.58 (2.23)	4.03 (4.2)	3.6 (4.24)	1.67 (1.02)	0.0008
Creatinine, mg/dL	1.27 (0.96)	1.09 (0.89)	1.06 (0.59)	1.09 (0.65)	1.28 (0.44)	0.96 (0.21)	0.0801
AST, U/L	41.71 (18.01)	73.48 (119.23)	63.09 (65.57)	73.58 (103.5)	46 (26.14)	54.68 (51.09)	0.3755
ALT, U/L	27.45 (16.35)	40.9 (39.86)	39.56 (38.93)	41.46 (47.24)	30 (9.74)	55.53 (124.29)	0.3760
INR	1.38 (0.28)	1.53 (0.69)	1.4 (0.27)	1.5 (0.45)	1.49 (0.43)	1.25 (0.21)	0.0125
LDH, U/L	163.15 (63.53)	256.65 (162.44)	271.58 (172.76)	210.57 (62.79)	313.86 (132.32)	226.92 (54.02)	0.0004
PTT, s	42.54 (11.44)	46.97 (18.99)	45.86 (16.58)	46.69 (15.09)	46 (16.25)	43.11 (23.18)	0.3581
Quick, %	62.83 (15.6)	58.81 (17.1)	60.61 (12.96)	59 (16.4)	61.5 (21.46)	73.74 (14.94)	0.0057
Sodium, mmol/L	136.7 (4.88)	138.84 (4.27)	139.34 (2.99)	137.92 (4.87)	140.56 (3.71)	140.39 (2.33)	0.0003
Urea, mg/dL	21.79 (15.17)	20.8 (15.74)	17.36 (7.76)	16.94 (9.1)	26.75 (22.41)	15.62 (4.27)	0.1490
PPG, mmHg	8.64 (3.34)	8.56 (3.25)	8.89 (3.21)	8.04 (3.59)	7.6 (3.03)	9.95 (3.34)	0.4051+

* Type III *p*-value for a time effect estimated in a mixed linear model with repeated measurement and a first order autoregressive covariance structure. + PPG was tested with a variance components covariance structure, due to computational instability. Abbreviations: ALT, Alanine Aminotransferase; AST, Aspartate Aminotransferase; FU, follow-up; INR, International Normalized Ratio; LDH, Lactate dehydrogenase; MELD, Model of End Stage Liver Disease; PPG, portal pressure gradient; PTT, Partial Thromboplastin Time; SD, standard deviation; TIPS, transjugular intrahepatic portosystemic shunt.

**Table 3:** Baseline and follow-up liver volume regarding TIPS insertion (mean (SD)).

	Baseline	FU 1	FU 2	FU 3	FU 4	FU 5	*p*-Value *
		90–180 d	180–365 d	365–545 d	545–730 d	>730 d	
TLV	1603.66 (561.4)	1446.52 (500.03)	1427.53 (544.12)	1432.83 (510.6)	1149.18 (364.63)	1373.71 (702.68)	<0.0001
LLV	613.31 (283.67)	571.31 (353.7)	526.64 (286.17)	606.11 (304.59)	460.19 (181.63)	488.87 (331.78)	0.0102
RLV	904.17 (382.13)	781.14 (323.07)	818.57 (290.14)	766.77 (298.45)	647.5 (245.71)	810.49 (342.41)	<0.0001
TLV/weight	20.6 (7.76)	18.43 (7)	17.79 (7.12)	17.25 (5.66)	18.19 (8.79)	16.93 (8.55)	<0.0001

* Type III *p*-value for a time effect estimated in a mixed linear model with repeated measurement and a first order autoregressive covariance structure. Abbreviations: FU, follow-up; LLV, left liver volume; RLV, right liver volume; SD, standard deviation; TIPS, transjugular intrahepatic portosystemic shunt; TLV, total liver volume.

**Table 4:** Correlation analysis of total liver volume (TLV) and liver function at first follow-up period (FU1: 90–180 d).

Correlation *\*p*-Value	TLV	TLV/Weight	MELD	Albumin	Bilirubin	Creatinine	AST	ALT	INR	LDH	PTT	Quick	Sodium	Urea
TLV	./.	0.00065	0.1973	0.74895	0.03839	0.44190	0.48834	0.64177	0.51723	0.67516	0.81028	0.46601	0.58287	0.88812
TLV/weight	0.69624	./.	0.0649	0.45499	0.04249	0.85433	0.61763	0.58616	0.26867	0.60208	0.78863	0.24511	0.80954	0.88729
MELD	−0.28579	−0.42046	./.	0.2530	<0.0001	0.0063	0.1803	0.7223	<0.0001	0.6677	0.0100	<0.0001	0.9844	0.0006
Albumin	0.10934	0.26748	−0.31485	./.	0.74289	0.01812	0.72696	0.51659	0.36531	0.57033	0.59542	0.51607	0.14692	0.28611
Bilirubin	−0.44413	−0.45760	0.75902	0.09254	./.	0.82206	0.00303	0.01641	0.00016	0.94956	0.00107	0.00005	0.99360	0.01020
Creatinine	0.17280	0.04386	0.4742	−0.59971	−0.04139	./.	0.48094	0.19296	0.66223	0.22505	0.41006	0.76493	0.65456	0.01296
AST	−0.15593	−0.11889	0.24702	0.09848	0.51506	−0.13144	./.	0.00012	0.09792	0.40718	0.09001	0.10339	0.27817	0.25259
ALT	−0.10783	0.13339	0.06768	0.18943	0.43458	−0.24445	0.65256	./.	0.16737	0.03503	0.31801	0.29051	0.35454	0.91387
INR	−0.14585	−0.25979	−0.67927	−0.25179	0.61828	−0.08029	0.30267	0.16737	./.	0.55882	<0.0001	<0.0001	0.80411	0.15309
LDH	0.11807	0.15978	0.10233	−0.18246	−0.01512	0.28394	0.19616	0.03503	0.13904	./.	0.16707	0.53069	0.82577	0.43791
PTT	0.05431	−0.06401	0.44845	−0.14928	0.55158	−0.15079	0.30968	0.31801	0.81249	0.32138	./.	<0.0001	0.44752	0.07011
Quick	0.16393	0.27249	−0.67927	0.18206	−0.65460	0.05501	−0.29807	−0.19951	−0.98577	−0.14900	−0.79421	./.	0.88089	0.10789
Sodium	0.12387	−0.05756	0.00359	0.39334	−0.00148	−0.08223	−0.20104	−0.17516	−0.04564	−0.05257	−0.13915	0.02758	./.	0.95956
Urea	0.03826	−0.04176	0.70034	−0.40000	0.56021	0.54497	0.26838	−0.02746	0.33170	0.22567	0.41327	−0.37043	0.01212	./.

* Spearman’s correlation coefficient in the lower triangle of the table, *p*-values in the upper triangle of the table (grey background). Abbreviations: ALT, Alanine Aminotransferase; AST, Aspartate Aminotransferase; FU, follow-up; INR, International Normalized Ratio; LDH, lactate dehydrogenase; MELD, Model of End-Stage Liver Disease; PPG, portal pressure gradient; PTT, Partial Thromboplastin Time; SD, standard deviation; TIPS, transjugular intrahepatic portosystemic shunt; TLV, total liver volume.

**Table 5:** Regression coefficients for the explorative model for total liver volume and time.

Effect	Estimate	Standard Error	Pr > |t|	Lower	Upper
Intercept	47.1441	82.8292	0.5711	−118.05	212.34
Time (days since TIPS)	1.5689	0.9389	0.1015	−0.3210	3.4588
Volume before TIPS	0.9403	0.04938	<0.0001	0.8418	1.0387
Time × volume before TIPS	−0.00143	0.000617	0.0251	−0.00267	−0.00019
Time^2^ × volume	2.031 × 10^−6^	2.346 × 10^−6^	0.3910	−2.69 × 10^−6^	6.75 × 10^−6^
Time^2^	−0.00226	0.003483	0.5195	−0.00927	0.00475
Time^3^ × volume	−14 × 10^−11^	0	<0.0001	.	.
Time^3^	−3.58 × 10^−7^	2.968 × 10^−6^	0.9045	−6.33 × 10^−6^	5.615 × 10^−6^

Time was included in this model as a random effect with a spatial power covariance structure. For this model higher order polynomials (order 3) for the fixed time effect and the time x volume (before TIPS) interactions were selected. Abbreviations: TIPS, transjugular intrahepatic portosystemic shunt.

**Table 6:** Regression coefficients for the explorative model for TLV/weight ratio and time.

Effect	Estimate	Standard Error	Pr > |t|	Lower	Upper
Intercept	2.1375	0.06172	<0.0001	2.0143	2.2607
Time (days since TIPS)	0.000454	0.000630	0.4749	−0.00082	0.000172
TLV/weight before TIPS	0.03932	0.002835	<0.0001	0.03366	0.04498
Time x TLV/weight before TIPS	−0.00004	0.000032	0.1792	−0.00011	0.00002
Time^2^ × TLV/weight	4.858 × 10^−8^	0	<0.0001	.	.
Time^2^	−4.64 × 10^−7^	2.45 × 10^−6^	0.8506	−5.4 × 10^−6^	4.473 × 10^−6^
Time^3^ × TLV/weight	3.86 × 10^−11^	0	<0.0001	.	.
Time^3^	−1.11 × 10^−9^	0	<0.0001	.	.

Time was included in this model as a random effect with a spatial power covariance structure. For this model higher order polynomials (order 3) for the fixed time effect and the time x volume (before TIPS) interactions were selected. Abbreviations: TIPS, transjugular intrahepatic portosystemic shunt; TLV, total liver volume.

**Table 7:** Regression coefficients for the final multivariate model for the development of total liver volume.

Effect	Estimate	Standard Error	Pr > |t|	Lower	Upper
Intercept	408.08	124.74	0.0017	159.10	657.06
Time (days since TIPS)	−0.7438	0.1730	<0.0001	−1.0900	−0.3976
INR before TIPS	−220.14	90.5786	0.0178	−400.94	−39.3451
Time × INR	0.3992	0.1203	0.0015	0.1586	0.6399
TLV before TIPS (ml)	0.8909	0.04062	<0.0001	0.8098	0.9720

Estimated with a linear mixed model and spatial power covariance structure for repeated measurements. Model characteristics: -2 Log-likelihood = 1802; Covariance parameter SP(POW) = 0.8113 (SE: 16.23). Abbreviations: INR, International Normalized Ratio; SP(POW), spatial power; TIPS, transjugular intrahepatic portosystemic shunt.

## Data Availability

Analyzed data are stored at the Clinic for Radiology, University Hospital in Muenster, Germany, and are available on reasonable request if not already included in this article.
